# Concurrent Validity and Between-Session Reproducibility of Agreement of GPath for Mean Propulsive Velocity Assessment During the Bench Press Exercise

**DOI:** 10.3390/sports14070263

**Published:** 2026-06-26

**Authors:** Alejandro Soler-López, Elena López-Martínez, Rubén Toledo-Pozuelo, Carlos D. Gómez-Carmona

**Affiliations:** 1 OncoSport Never Surrender Foundation, 30005 Murcia, Spain; investigacion@neversurrenderf.org (A.S.-L.); rubencasas@neversurrenderf.org (R.T.-P.); 2 BioVetMed & SportSci Research Group, University of Murcia, 30001 Murcia, Spain; 3 Faculty of Sport Science, UCAM Catholic University of Murcia, 30107 Murcia, Spain; 4 Physical Activity and Sports Area, Department of General and Specific Didactics, University of Alicante, 03690 San Vicente del Raspeig, Spain; 5 Research Group of Training, Physical Activity and Sports Performance (ENFYRED), University of Zaragoza, 20020 Huesca, Spain

**Keywords:** velocity-based training, inertial measurement unit, linear position transducer, concurrent validity, between-session reproducibility

## Abstract

Growing use of inertial measurement units (IMUs) in velocity-based training has outpaced the evidence supporting their accuracy, making device-specific validation an essential aspect before adoption in research or practice. This study evaluated the concurrent validity of the GPath 6-axis inertial sensor against the Chronojump linear position transducer (LPT) for mean propulsive velocity (MPV) measurement during the bench press exercise. Twelve physically active males performed repetitions at 20%, 40%, 60%, and 80% of one-repetition maximum (1RM) across two separate testing sessions. Concurrent validity was evaluated using Pearson’s correlation, concordance correlation coefficient (*CCC*), Bland–Altman analysis, typical error, and coefficient of variation (*CV*). The GPath systematically overestimated MPV relative to the LPT at all loading conditions (*p* < 0.001), with bias decreasing from 0.253 m/s (20% 1RM) to 0.091 m/s (80% 1RM). *CV* remained low and consistent across conditions (5.12–5.98%). *CCC* was trivial > 1 m/s (20–40% 1RM: 0.079–0.056; combined: 0.331) and moderate < 1 m/s (60–80% 1RM: 0.430–0.433; combined: 0.833), with an overall *CCC* of 0.877. Between-session reproducibility of agreement was high (Session 1: *CCC* = 0.878; Session 2: *CCC* = 0.877), with between-session bias differences not exceeding 0.026 m/s. Despite its systematic overestimation, the GPath showed a reproducible bias structure between sessions, which may indicate utility for longitudinal within-athlete monitoring of relative MPV changes. Although, it cannot be recommended for absolute velocity measurement or 1RM estimation without prior correction.

## 1. Introduction

Velocity-based training (VBT) has become established as a scientific method for prescribing and monitoring resistance training. This method offers objective regulation of training intensity through real-time barbell kinematics [1]. Movement velocity has been widely used as an objective index of neuromuscular fatigue during strength training [2,3]. Therefore, the progressive adoption of VBT in both elite sport and health-oriented exercise settings has driven the development of accurate, portable, and affordable devices [4]. Early VBT implementations relied on linear position transducers (LPTs) such as wire-based encoders. These devices offer high resolution and accuracy but require considerable infrastructure and restrict their use to vertically constrained barbell movements [5]. This limitation spurred the development of portable alternatives based on inertial measurement units (IMUs) that estimate barbell velocity by integrating acceleration data using proprietary algorithms [6]. Regardless of the technological format, any measurement instrument must demonstrate adequate concurrent validity against an established criterion before its implementation [7,8], a standard also reflected in sports technology quality frameworks [9].

The validation of IMU-based velocity devices against LPTs has produced heterogeneous results that reflect the device-specific nature of measurement error and the influence of loading conditions on agreement. Clemente et al. [10] reviewed 22 studies covering eight commercially available IMU models and reported *ICC* values ranging from 0.27 to 0.99 depending on device, exercise, and velocity range, with *CV* values from 2.1% to over 35%. Pérez-Castilla et al. [11] tested seven devices simultaneously during the bench press and found that LPTs outperformed IMUs in both concurrent validity and test–retest reliability, with systematic MPV overestimation being a common characteristic of accelerometer-based systems. Several authors have applied VBT in real-world settings, noting the affordability and practicality of available devices [4,12]. This has encouraged the search for low-cost alternatives without compromising measurement accuracy.

In this regard, several studies have reported device-specific validity data for individual IMU models. Regarding the PUSH Band, Courel-Ibáñez et al. [13] found *SEE* values of 0.135 m/s during bench press and 0.091 m/s during squat against the Chronojump, with *ICC* values of 0.97 for both exercises. Lake et al. [14] further confirmed these findings during bench press at moderate and heavy loads, reporting *ICC* values between 0.93 and 0.97. For the Wimu RealTrack Systems, Pino-Ortega et al. [15] reported bias values of 0.011 m/s and *R*^2^ of 0.99 during leg extension, while Muyor et al. [16] found *CV* values > 5% in back squat exercise. Regarding the VmaxPro^®^, Held et al. [17] reported *ICC* values of 0.91–0.96 and limits of agreement below 0.12 m/s during squats and hip thrusts. Feuerbacher et al. [18] found high correlations against both Vicon and Chronojump (*R*^2^ > 0.93), but also a consistent mean velocity overestimation of around 0.06 m/s, a pattern also observed for the EnodePro® during bench press and squat [19]. Dragutinovic et al. [20] and van den Tillaar et al. [21] similarly reported that IMU validity is context-dependent, with errors shaped by movement velocity, exercise type, and device-specific characteristics. Taken together, these findings indicate that sensor characteristics and algorithmic processing play a decisive role in measurement accuracy.

The GPath 6-axis inertial sensor (GPath Technologies, Gdańsk, Poland) is a recently commercialized IMU designed for MPV estimation during free-weight resistance exercises. However, no previous research has evaluated the validity of this device. Given the device- and load-dependent nature of IMU measurement error described above, including effects attributed to acceleration signal integration [21,22], this gap is particularly relevant. The Chronojump (Chronojump Boscosystem, Barcelona, Spain) is a cable-extension linear position transducer previously validated against the T-Force linear velocity transducer [23] and a 3D optoelectronic system [11]. Therefore, the present study aimed: (1) to determine the concurrent validity (agreement) of the GPath 6-axis inertial sensor against the Chronojump LPT for MPV assessment during the bench press across four loading intensities (20%, 40%, 60%, and 80% of 1RM); and (2) to examine the between-session reproducibility of this agreement structure across two separate testing days.

## 2. Materials and Methods

### 2.1. Study Design

A cross-sectional concurrent validity and between-session reproducibility study was designed to compare MPV measurements recorded simultaneously by an LPT (Chronojump Boscosystem, Barcelona, Spain) and an IMU (GPath Technologies, Gdańsk, Poland) during the bench press exercise at four relative loading conditions. All measurements were conducted in an indoor laboratory at a controlled ambient temperature of 20–22 °C and relative humidity of 45–55%. Testing was performed on a Smith machine, using a standard 20-kg Olympic bar, with participants lying supine and feet flat on the floor throughout all repetitions. The study followed the methodological framework for instrument validation in sport science proposed by Ibáñez and Feu [24], evaluating measurement agreement across the full range of exercise intensities tested.

### 2.2. Participants

Twelve physically active males were recruited to participate in this study (*M* ± *SD*; age: 26.8 ± 2.1 years; height: 178.4 ± 5.6 cm; body mass: 80.2 ± 9.3 kg; BMI: 25.2 ± 2.1 kg/m^2^). This sample size is consistent with previous concurrent validity studies of IMU-based velocity devices, which have typically employed samples of 10 to 20 trained participants without a formal a priori power analysis [11,13,14,17]. Inclusion criteria were: (a) minimum two years of supervised bench press training experience; (b) absence of musculoskeletal injuries in the six months preceding data collection; and (c) no use of ergogenic aids in the 48 h prior to the test session. All participants received written and verbal information about the study objectives and procedures and provided written informed consent prior to enrolment.

The study was approved by the Ethics Committee of the University of Murcia (Reg. code: 2061/2018, Approval Date: 20 November 2018) and conducted in accordance with the ethical principles of the Declaration of Helsinki [25]. The estimated 1RM ranged from 66.8 to 110.0 kg (*M* ± *SD*: 88.5 ± 17.0 kg). Based on these individual values, absolute loads at each relative intensity were: 20% 1RM: 17.6 ± 3.4 kg (13.2–22.0 kg); 40% 1RM: 35.3 ± 6.8 kg (26.4–44.0 kg); 60% 1RM: 54.0 ± 8.8 kg (42.0–66.0 kg); and 80% 1RM: 70.5 ± 13.7 kg (52.8–88.0 kg).

### 2.3. Instruments

**Figure 1:**
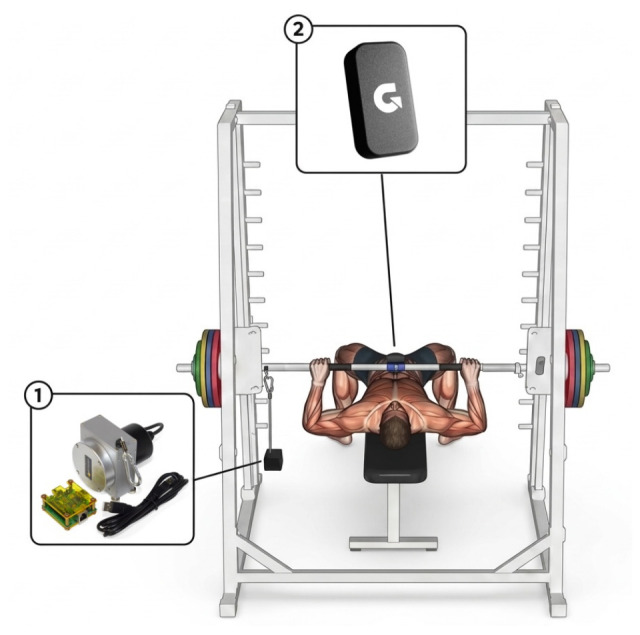
Measurement system configuration: Chronojump LPT (1) and GPath sensor (2).

Figure 1 illustrates the experimental setup, showing the positioning of the measurement devices relative to the participant during the bench press protocol.

**Chronojump LPT.** The Chronojump (Linear encoder kit, Chronojump Boscosystem, Barcelona, Spain) is a cable-extension linear position transducer that records barbell displacement at 1000 Hz sampling frequency. The device provides real-time output of mean velocity, MPV, and peak velocity. Its validity and reliability for VBT have been demonstrated against the T-Force LPT across multiple resistance exercise conditions in older adults [23], and against a 3D optoelectronic system during the bench press [11]. For this study, the transducer cable was vertically attached to the barbell following the manufacturer’s standard procedure. The Chronojump served as the reference device.

**GPath 6-axis inertial sensor.** The GPath (GPath Technologies, Gdańsk, Poland) is a portable IMU designed to estimate MPV during free-weight resistance exercises via proprietary algorithms. The GPath integrates a 6-axis inertial sensor (3-axis accelerometer and 3-axis gyroscope) and communicates via Bluetooth 5.0. Beyond these hardware specifications, the manufacturer does not publicly disclose sampling frequency, filtering procedures, or the proprietary velocity calculation algorithm. An integrated neodymium magnet enables tool-free attachment to any standard barbell. Before each session, the device was manually calibrated on a flat, horizontal, metallic-element-free surface following the manufacturer’s instructions. The neodymium magnet was detached during calibration to avoid distortion of the internal magnetic reference field and reattached upon completion.

### 2.4. Variables and Procedure

The dependent variable for both devices was mean propulsive velocity (MPV). MPV is defined as the average barbell velocity during the propulsive phase of the concentric contraction. The propulsive phase is operationally defined as the portion of the concentric stroke in which the net muscular force applied to the barbell exceeds its gravitational load (9.8 m/s^2^). By excluding the terminal braking phase, in which the lifter decelerates the bar to prevent uncontrolled release and whose duration varies across individuals, MPV provides a more stable and load-specific kinematic index than mean velocity [3]. Each repetition was recorded simultaneously by the Chronojump LPT and GPath sensor, with the initiation of the concentric phase serving as the common temporal reference point for both devices, and computed MPV following this same operational definition [26].

The study comprised three sessions, each separated by a minimum of 72 h. During the first session, participants were familiarized with the testing procedures and both measurement devices. All participants performed the bench press with a standard prone grip and an individually standardized grip width (1.5 × biacromial distance) in a Smith machine constraining the barbell to a fixed vertical displacement path to ensure valid measurements from both devices. The Chronojump LPT was positioned inside the Smith machine frame to the left of the barbell to minimize cable vibration, with the cable attached vertically to the bar. The GPath sensor was magnetically fixed to the center of the barbell on its upper surface, perpendicular to the bar axis and oriented upward during the lifting phase to optimize alignment with the vertical axis, as illustrated previously in Figure 1.

In the second and third sessions, 1RM was estimated following the velocity-load profiling method of González-Badillo and Sánchez-Medina [2]. It uses single sets with progressively heavier loads until MPV dropped below 0.60 m/s, from which 1RM was extrapolated via individual linear regression. Then, participants performed three repetitions at four loads (20%, 40%, 60%, and 80% of 1RM), applied in ascending order following standard incremental loading protocols [2], as higher-velocity lighter loads are more sensitive to fatigue-induced decrements than heavier loads. All repetitions were performed with maximal intentional concentric velocity [27], with 3-min recovery between loads.

Before each loading set, a confirmation repetition verified that the MPV matched the expected value for that intensity based on the load–velocity relationship (20% 1RM: ≈1.33 m/s; 40% 1RM: ≈1.11 m/s; 60% 1RM: ≈0.77 m/s; 80% 1RM: ≈0.46 m/s) [26]. If the deviation exceeded ±0.05 m/s, a 60-s rest was given, and the confirmation was repeated modifying the load until the target was met. The three repetitions of each load were performed without velocity constraints, naturally reflecting fatigue-induced decrements within the set. At the end of each session, data were exported from Chronojump software (version 2.6.0 for Apple Silicon; Chronojump Boscosystem, Barcelona, Spain) and GPath smartphone application (version 2.0.13 for iOS; GPath Technologies, Gdańsk, Poland) to an Excel spreadsheet for further analysis.

### 2.5. Statistical Analysis

Statistical analyses were performed in Python (v3.11). Each repetition was used as the unit of analysis, consistent with the approach adopted in comparable concurrent validity studies of VBT devices [11,13,17,19,20,21], and reflecting the real-time, repetition-level feedback that coaches and athletes receive from these devices in applied settings. The normality and homoscedasticity of data distributions were assessed using the Shapiro–Wilk and Levene test, confirming that both assumptions were met at all loading conditions (all *p* > 0.05).

Concurrent validity of the GPath sensor relative to the Chronojump LPT was evaluated using: (a) Pearson’s correlation coefficient (*r*) to quantify the linear association between devices; (b) the concordance correlation coefficient (*CCC*), which simultaneously evaluates precision (correlation component) and accuracy (bias component) and is specifically suited to method-comparison studies, with 95% confidence intervals calculated via the Z-transformation, using the exact asymptotic variance formula derived by Lin [28]; (c) Bland–Altman analysis [29], reporting the systematic bias (mean difference, GPath − sensor − Chronojump LPT) and the 95% limits of agreement (*LoA* = bias ± 1.96 × SD of differences), with heteroscedasticity assessed via Spearman’s correlation between absolute differences and condition means [30]; (d) the typical error (*TE* = SD_diff_/√2), a measure of random measurement error between two methods [31]; and (e) the coefficient of variation (*CV*% = [SD_diff_/MeanLPT] × 100). A paired-samples *t*-test assessed whether the observed bias differed significantly from zero. The magnitude of *r* and *CCC* was interpreted according to Hopkins [8] as trivial (<0.10), small (0.10–0.30), moderate (0.30–0.50), large (0.50–0.70), very large (0.70–0.90), or nearly perfect (>0.90).

To further examine the influence of velocity range on agreement, loading conditions were additionally grouped above and below the 1 m/s threshold commonly used in veloci-ty-based training practice. Finally, to examine the consistency of validity across sessions, all validity analyses were repeated independently for session two and session three. Between-session agreement was then evaluated by comparing the bias, *LoA*, *CCC*, and *CV* values obtained in each session. All analyses were conducted both per loading condition and overall. Statistical significance was set at α = 0.05.

## 3. Results

### 3.1. Descriptive and Concurrent Validity Statistics

Concurrent validity statistics are presented in Table 1. The GPath sensor systematically overestimated MPV relative to the LPT across all loading conditions (*p* < 0.001). Systematic bias decreased from 0.253 ± 0.078 m/s at 20% 1RM to 0.091 ± 0.026 m/s at 80% 1RM. All limits of agreement remained positive across the full loading range. Pearson correlation increased from 0.501 at 20% 1RM to 0.926 at 80% 1RM. *CCC* ranged from 0.056 at 40% 1RM to 0.432 at 80% 1RM. The CV was consistent across all conditions, ranging from 5.12% to 5.98%. Overall, *r* was 0.988, *CCC* was 0.877, (95% CI: 0.858, 0.892), and bias was 0.167 ± 0.090 m/s.

To further examine the influence of velocity range, loading conditions were grouped according to the 1 m/s threshold commonly used in VBT practice. For repetitions > 1 m/s (20% and 40% 1RM), systematic bias was 0.238 ± 0.069 m/s (*LoA*: 0.102–0.374 m/s), *r* = 0.870 (*p* < 0.001), *CCC* = 0.331 (95% CI: 0.288, 0.369), *TE* = 0.049 m/s, and *CV* = 5.78%. For repetitions < 1 m/s (60% and 80% 1RM), systematic bias was 0.095 ± 0.034 m/s (*LoA*: 0.029–0.161 m/s), *r* = 0.979 (*p* < 0.001), *CCC* = 0.833 (95% CI: 0.804, 0.857), *TE* = 0.024 m/s, and *CV* = 5.52%.

### 3.2. Correlation Analysis by Loading Condition

**Figure 2:**
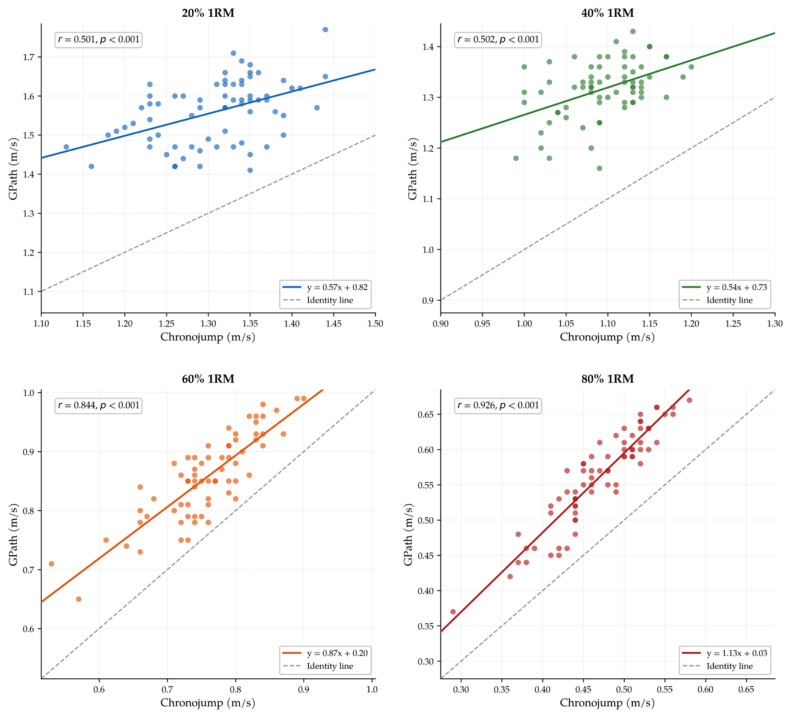
Correlation plots between Chronojump LPT (*x*-axis) and GPath sensor (*y*-axis) for each percentage of 1RM. The dashed black line represents the identity line (*y* = *x*); the solid-colored line represents the ordinary least-squares regression line.

Figure 2 presents the scatter plots with regression and identity lines (*y* = *x*) for each loading condition. The overall Pearson correlation was excellent (*r* = 0.988, *p* < 0.001), reflecting the wide velocity range of the full dataset (~0.29–1.60 m/s). Per-load correlations improved progressively with intensity: from *r* = 0.501 at 20% and *r* = 0.502 at 40% 1RM to *r* = 0.844 and *r* = 0.926 at 60% and 80% 1RM, respectively.

### 3.3. Bland–Altman Analysis

**Figure 3:**
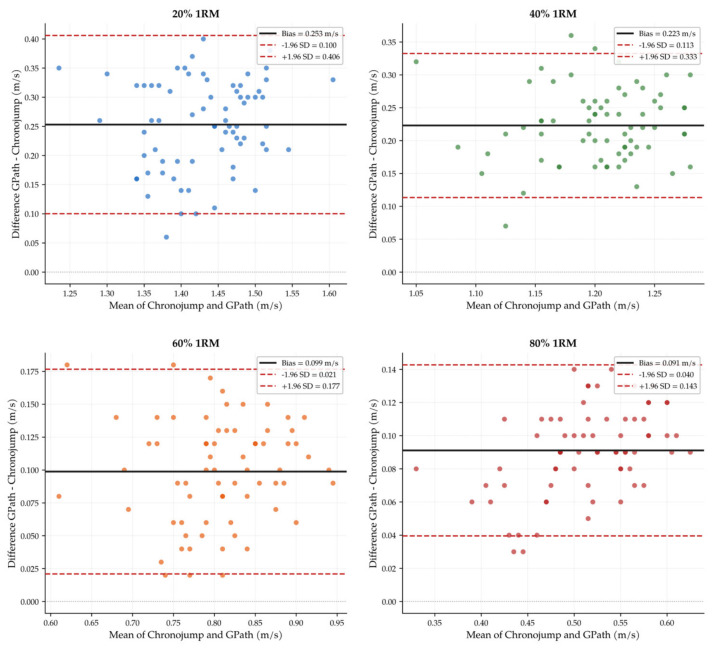
Bland–Altman plots of the difference between GPath and Chronojump (*y*-axis) against their mean (*x*-axis) by loading condition. The solid black line represents the systematic bias; the dashed red lines represent the 95% limits of agreement (bias ± 1.96 SD).

Bland–Altman plots are presented in Figure 3. A positive and statistically significant bias was observed in all conditions (*p* < 0.001). Bias decreased with increasing load from 0.253 m/s at 20% 1RM to 0.091 m/s at 80% 1RM. The narrowest LoA were observed at 80% 1RM, indicating the most consistent agreement between devices at the lowest MPV. No significant heteroscedasticity was observed at any loading condition, as evidenced by Spearman correlations between absolute differences and means (*r*_s_ < 0.147; *p* > 0.119). Regression analysis of differences against means revealed no significant proportional bias at 20%, 40%, or 60% 1RM (slopes = 0.163, 0.089, and 0.035; *R*^2^ = 0.020, 0.006, and 0.004; all *p* > 0.05). A significant proportional bias was identified at 80% 1RM (*slope* = 0.203, *R*^2^ = 0.214, *p* < 0.001) and overall (*slope* = 0.195, *R*^2^ = 0.607, *p* < 0.001). 

### 3.4. Between-Session Reproducibility of Agreement

The systematic overestimation of MPV by the GPath sensor relative to the LPT was consistent across sessions at all velocity conditions (*p* < 0.001 in all conditions), with between-session bias differences not exceeding 0.001 m/s at any velocity group. *CCC* values were virtually identical between sessions, both above 1 m/s (Session 1: 0.322; Session 2: 0.340) and below 1 m/s (Session 1: 0.827; Session 2: 0.840), and overall *CCC* remained stable (Session 1: 0.880; Session 2: 0.874). *CV* was consistently low and stable across sessions in both velocity groups (range: 5.39–5.93%) (see Table 2 for more details). 

## 4. Discussion

The main finding of this study is that the GPath 6-axis inertial sensor systematically overestimates MPV relative to the Chronojump across all loading intensities during the bench press, with bias decreasing monotonically from 0.253 m/s at 20% 1RM to 0.091 m/s at 80% 1RM. This pattern is consistent with the systematic MPV overestimation reported by Pérez-Castilla et al. [11] for the majority of IMU devices evaluated during the bench press and aligns with the findings by van den Tillaar et al. [21] that accelerometer-based systems tend to accumulate greater errors at lower loads associated with higher movement velocities. More recently, Behrmann et al. [19] confirmed a similar load-dependent overestimation pattern for the EnodePro during bench press and squat, with mean absolute errors ranging from 0.076 to 0.123 m/s, further illustrating that systematic overestimation is a recurring finding across IMU-based devices.

The random error component of the GPath sensor was consistently low across all conditions (*CV* = 5.12–5.98%), following the criterion proposed by Muyor et al. [16] that *CV* < 10% represents an indicator of acceptable agreement, and subsequently adopted across the VBT device validation literature [4,10]. However, while these *CV* values in-dicate acceptable random error consistency, they should be interpreted alongside the systematic bias observed, particularly at lighter loads where overestimation exceeded 0.223 m/s. The degree of agreement achieved by IMU devices varies considerably depending on device model and exercise evaluated, as illustrated by the near-perfect agreement reported for the WIMU PRO during leg extension (*bias* = 0.011 m/s; *r* = 0.999) [15] and the low *CV* values observed for the VmaxPro [17], WIMU PRO [16] and EnodePro [19], compared with the higher *CV* of the PUSH Band (CV > 9%) [13] and Beast Sensor (*CV* > 35%) [10]. 

Agreement improved progressively with increasing load, though per-load *CCC* values remained trivial-to-moderate across all loading conditions. *CCC* was trivial at 20% and 40% 1RM (0.079 and 0.056) and only moderate at 60% and 80% 1RM (0.429 and 0.432), while Pearson r ranged from large (0.501 at 20% 1RM) to nearly perfect (0.926 at 80% 1RM), following the magnitude thresholds proposed by Hopkins et al. [8]. The very high overall Pearson correlation (r = 0.988) reflects association across a broad pooled velocity range rather than agreement, as correlation is known to depend on this range regardless of the actual concordance between methods [29]. When loading conditions were grouped by the 1 m/s threshold, *CCC* was 0.331 above 1 m/s and 0.833 below 1 m/s. While the latter value is more favorable, systematic bias remained present below 1 m/s (0.095 m/s), and the wider between-subject velocity range in the grouped analysis is known to influence agreement indices [28,29]. The stable *CV* across all loading conditions confirms that the low per-load *CCC* values primarily reflect the dominance of systematic bias over between-subject variability [28]. 

Regression analysis confirmed no significant proportional bias at 20%, 40%, or 60% 1RM. Although heteroscedasticity (dispersion of differences) and proportional bias (magnitude-dependence of the bias itself) are distinct properties, a significant proportional bias was found at 80% 1RM (*R*^2^ = 0.214); nonetheless, the systematic bias remained predominantly fixed across loading conditions, suggesting that a load-specific correction could reduce measurement errors [32]. The higher *R*^2^ obtained when pooling all loading conditions (*R*^2^ = 0.607) likely reflects the aggregation of four discrete bias magnitudes rather than a true proportional relationship within any single condition, consistent with the known inflation of association measures when heterogeneous subgroups are combined [28,29].

The between-session analysis revealed a highly reproducible agreement structure across testing days, with *CCC* remaining stable both above 1 m/s (Session 1: 0.322; Session 2: 0.340) and below 1 m/s (Session 1: 0.827; Session 2: 0.840), bias differences not exceeding 0.001 m/s at any velocity group, and *CV* consistently below 6% across both sessions. Overall, these findings confirm that the systematic overestimation is a stable device characteristic, consistent with reproducible error structures reported for comparable IMU devices [17,19,20]. As noted by Feuerbacher et al. [18], a stable error structure may allow within-athlete monitoring of relative velocity changes even in the absence of absolute accuracy, provided the systematic bias is not used for direct device substitution [9,28,29].

The practical implications of the observed systematic bias are particularly relevant for VBT load prescription. Based on the load–velocity relationship established for the bench press [26], where a velocity change of approximately 0.07–0.09 m/s corresponds to a 5% difference in relative load, the bias values observed above 1 m/s (0.223–0.253 m/s) translate into load prescription errors of approximately 12–18% 1RM, while below 1 m/s (0.091–0.099 m/s) errors correspond to approximately 5–7% 1RM. Although these estimates are derived from a well-established load–velocity relationship [26], they should be regarded as approximate given the documented inter-individual variability in this relationship [33]. These magnitudes exceed the practically acceptable threshold for MPV error in VBT contexts of approximately 0.05–0.07 m/s (~3–5% 1RM) [2,3], a threshold that is only approached by the GPath sensor at 80% 1RM (*bias* = 0.091 m/s). This loading-dependent pattern of agreement mirrors findings for the VmaxPro [18,20], the PUSH Band [13,14] and the EnodePro [19], all of which showed lower agreement at lighter, higher-velocity loading conditions.

To the best of our knowledge, this is the first study to examine the concurrent validity of the GPath sensor against a validated LPT across multiple loading intensities and two separate testing sessions during the bench press exercise. This provides novel and practically relevant information for researchers and practitioners considering this device for velocity-based training monitoring. Nevertheless, several limitations should be acknowledged. The sample was restricted to physically active young males, which constrains the generalizability of findings to other populations such as female athletes, older adults, or individuals with different training backgrounds. Although the sample size (*n* = 12) is consistent with prior IMU validation studies, it may limit the precision of load-specific CCC estimates. Additionally, repetitions were nested within the 12 participants rather than fully independent, which may have affected the precision of the reported confidence intervals and significance tests. All testing was conducted under controlled laboratory conditions using the bench press exercise exclusively, limiting generalizability to field environments and other exercises. Although the ascending load order was methodologically justified, potential order effects cannot be entirely excluded. Finally, the absolute accuracy of the LPT as a reference device could not be independently verified in the absence of an independent motion-capture criterion. Future research should therefore prioritize the assessment of test–retest reliability of the GPath across sessions and environmental conditions, its sensitivity to detect session-to-session velocity decrements as indicators of neuromuscular fatigue, its validity across other exercises and populations, and the potential benefit of larger samples to improve the precision of load-specific agreement estimates.

## 5. Conclusions

The GPath demonstrates a systematic overestimation of MPV relative to the Chronojump LPT during the bench press across all loading intensities, with a consistent and reproducible error structure across two separate testing sessions. The random measurement error was low throughout (*CV* = 5.12–5.98%). CCC was trivial for loading conditions with velocities above 1 m/s (20% and 40% 1RM: 0.079 and 0.056, respectively) and moderate for loading conditions with velocities below 1 m/s (60% and 80% 1RM: 0.429 and 0.432, respectively), with an overall *CCC* of 0.877. The magnitude of the systematic bias precludes the direct substitution of LPT readings with uncorrected GPath values. Accordingly, the GPath cannot be recommended for absolute velocity measurement, load-velocity profiling, or non-maximal 1RM estimation without prior correction. However, because the systematic bias was directionally consistent and reproducible across sessions, session-to-session relative changes in MPV under fixed loading conditions would not be affected by this constant offset, provided the same device is used throughout monitoring. This may indicate a potential utility for longitudinal within-athlete monitoring, although this application remains to be confirmed by future research.

## Figures and Tables

**Table 1:** Descriptive statistics and concurrent validity indicators between Chronojump (reference) and GPath by loading condition and overall.

Variable	20% 1RM	40% 1RM	60% 1RM	80% 1RM	Overall
Chronojump LPT (m/s)	1.304 ± 0.073	1.092 ± 0.054	0.757 ± 0.070	0.469 ± 0.055	0.906 ± 0.327
GPath sensor (m/s)	1.557 ± 0.082	1.315 ± 0.058	0.856 ± 0.072	0.560 ± 0.067	1.074 ± 0.397
*r* (*p*)	0.501 (<0.001)	0.502 (<0.001)	0.844 (<0.001)	0.926 (<0.001)	0.988 (<0.001)
*CCC* [95% *CI*]	0.079 [0.043, 0.112]	0.056 [0.031, 0.079]	0.429 [0.340, 0.505]	0.432 [0.370, 0.485]	0.877 [0.858, 0.892]
*Bias* ± *SD* (m/s)	0.253 ± 0.078	0.223 ± 0.056	0.099 ± 0.040	0.091 ± 0.026	0.167 ± 0.090
Lower to Upper *LoA* (m/s)	0.100 to 0.406	0.113 to 0.333	0.021 to 0.177	0.040 to 0.143	−0.010 to 0.344
*TE* (m/s); *CV* (%)	0.055; 5.98	0.040; 5.12	0.028; 5.24	0.019; 5.61	0.064; 9.94
*t* (*p*)	18.080 (<0.001)	25.954 (<0.001)	14.665 (<0.001)	19.137 (<0.001)	19.081 (<0.001)

**Note.** Values are *M* ± *SD* for Chronojump LPT and GPath sensor. *r* = Pearson correlation; *CCC* = concordance correlation coefficient [95% confidence interval, *CI*]; *Bias* = mean difference (GPath sensor − Chronojump LPT); *LoA* = 95% limits of agreement; *TE* = typical error; *CV* = coefficient of variation; *t* = paired-samples *t*-test; *p* = significance.

**Table 2:** Between-session reproducibility of agreement between Chronojump LPT and GPath sensor, grouped by velocity threshold (>1 m/s and <1 m/s) and overall.

Variable	Session 1			Session 2		
	>1 m/s	<1 m/s	Overall	>1 m/s	<1 m/s	Overall
Chronojump LPT (m/s)	1.208 ± 0.127	0.606 ± 0.155	0.909 ± 0.333	1.190 ± 0.122	0.614 ± 0.162	0.904 ± 0.322
GPath sensor (m/s)	1.446 ± 0.134	0.701 ± 0.159	1.076 ± 0.402	1.428 ± 0.148	0.709 ± 0.170	1.071 ± 0.394
*r* (*p*)	0.863 (<0.001)	0.979 (<0.001)	0.987 (<0.001)	0.880 (<0.001)	0.979 (<0.001)	0.988 (<0.001)
*CCC* [95% CI]	0.322 [0.261, 0.375]	0.827 [0.783, 0.859]	0.880 [0.853, 0.901]	0.340 [0.279, 0.393]	0.840 [0.797, 0.872]	0.874 [0.847, 0.896]
*Bias* ± *SD* (m/s)	0.238 ± 0.069	0.095 ± 0.033	0.167 ± 0.090	0.238 ± 0.071	0.095 ± 0.035	0.167 ± 0.091
Lower to Upper *LoA* (m/s)	0.104 to 0.373	0.031 to 0.159	−0.009 to 0.343	0.099 to 0.376	0.027 to 0.163	−0.011 to 0.345
*TE* (m/s); *CV* (%)	0.048; 5.68	0.023; 5.39	0.063; 9.88	0.050; 5.93	0.025; 5.67	0.064; 10.04
*t* (*p*)	29.105 (<0.001)	24.145 (<0.001)	21.957 (<0.001)	28.389 (<0.001)	22.780 (<0.001)	21.828 (<0.001)

**Note.** Values are *M* ± *SD* for Chronojump LPT and GPath sensor. *r* = Pearson correlation; *CCC* = concordance correlation coefficient [95% confidence interval]; *Bias* = mean difference (GPath sensor − Chronojump LPT); *LoA* = 95% limits of agreement; *TE* = typical error; *CV* = coefficient of variation; *t* = paired-samples *t*-test; *p* = significance.

## Data Availability

The data presented in this study are available upon reasonable request to the corresponding author. The data are not publicly available due to privacy restrictions in accordance with the Spanish Organic Law 3/2018 of December 5, on the Protection of Personal Data and Guarantee of Digital Rights.

## Abbreviations

The following abbreviations are used in this manuscript:

IMU	Inertial measurement unit
LPT	Linear position transducer
MPV	Mean propulsive velocity
VBT	Velocity-based training
1RM	One-repetition maximum
CCC	Concordance correlation coefficient
CV	Coefficient of variation
LoA	Limits of agreement
TE	Typical error
SD	Standard deviation
BMI	Body mass index
